# Florally rich habitats reduce insect pollination and the reproductive success of isolated plants

**DOI:** 10.1002/ece3.3186

**Published:** 2017-07-11

**Authors:** Tracie M. Evans, Stephen Cavers, Richard Ennos, Adam J. Vanbergen, Matthew S. Heard

**Affiliations:** ^1^ NERC Centre for Ecology and Hydrology Wallingford UK; ^2^ Institute of Evolutionary Biology University of Edinburgh Edinburgh UK; ^3^ NERC Centre for Ecology and Hydrology Penicuik Edinburgh UK

**Keywords:** microsatellites, outcrossing, paternity analysis, pollen flow, pollen limitation, pollinator foraging, self‐fertilization, viability

## Abstract

Landscape heterogeneity in floral communities has the potential to modify pollinator behavior. Pollinator foraging varies with the diversity, abundance, and spatial configuration of floral resources. However, the implications of this variation for pollen transfer and ultimately the reproductive success of insect pollinated plants remains unclear, especially for species which are rare or isolated in the landscape. We used a landscape‐scale experiment, coupled with microsatellite genotyping, to explore how the floral richness of habitats affected pollinator behavior and pollination effectiveness. Small arrays of the partially self‐compatible plant Californian poppy (*Eschscholzia californica)* were introduced across a landscape gradient to simulate rare, spatially isolated populations. The effects on pollinator activity, outcrossing, and plant reproduction were measured. In florally rich habitats, we found reduced pollen movement between plants, leading to fewer long‐distance pollination events, lower plant outcrossing, and a higher incidence of pollen limitation. This pattern indicates a potential reduction in per capita pollinator visitation, as suggested by the lower activity densities and richness of pollinators observed within florally rich habitats. In addition, seed production reduced by a factor of 1.8 in plants within florally rich habitats and progeny germination reduced by a factor of 1.2. We show this to be a consequence of self‐fertilization within the partially self‐compatible plant, *E. californica*. These findings indicate that locally rare plants are at a competitive disadvantage within florally rich habitats because neighboring plant species disrupt conspecific mating by co‐opting pollinators. Ultimately, this Allee effect may play an important role in determining the long‐term persistence of rarer plants in the landscape, both in terms of seed production and viability. Community context therefore requires consideration when designing and implementing conservation management for plants which are comparatively rare in the landscape.

## 1 INTRODUCTION

Changes to the availability and diversity of floral resources through altered land use, including increased landscape fragmentation and simplification, can have considerable impacts on the structure, abundance, and diversity of pollinator communities (Potts et al., 2016; Senapathi et al., 2015; Vanbergen et al., 2013). With an estimated 87.5% of flowering plant species worldwide at least partly reliant upon pollinators for reproductive success and long‐term survival, this will have direct implications for plants (Ollerton, Winfree, & Tarrant 2011). By transferring conspecific pollen between plant individuals, pollinators not only facilitate seed production but have important effects on fitness and population genetic diversity by increasing outcrossing and the exchange of novel alleles (Frankham, 2005; Levin & Kerster, 1974; Mannouris & Byers, 2013).

Plant–pollinator interactions vary with plant population size, density, and habitat context (Essenberg, 2012; Mayer, Van Rossum & Jacquemart 2012). Habitats supporting a high abundance and species richness of flowering plants may either enhance or disrupt the transference of pollen to plants (Blaauw & Isaacs, 2014; Vanbergen et al., 2014). The outcome depends on pollinator visitation patterns, which are determined, in part, by the demography and characteristics of a species’ population relative to heterospecific co‐flowering plants (Essenberg, 2012). For instance, when at low floral densities, co‐flowering heterospecific plants can facilitate pollinator visitation to a plant population by enhancing the overall attractiveness of a floral patch (Rathcke, 1983). At high floral densities, co‐flowering heterospecific plants may result in inter‐specific competition for pollinators, which can reduce per capita visitation to a plant population, resulting in an insufficient supply of pollen that limits potential seed set (Ghazoul, 2006). Alternatively, although pollinators may prefer foraging on particular plant species (Chittka, Thomson, & Waser, 1999; Gegear & Laverty, 2005; Waser, 1986), such fidelity may be relaxed in communities with high floral diversity, increasing the potential for inter‐specific pollen transfer (Fontaine, Collin, & Dajoz, 2008). This has potential negative implications for plant reproduction. The supply of conspecific pollen to a plant can be reduced if it is lost during visitation to heterospecific plants (Wilcock & Neiland, 2002); moreover, the deposition of heterospecific pollen, by clogging the stigma and style of conspecific plants, can inhibit pollination (Holland & Chamberlain, 2007). Both lead to reduced pollination effectiveness and ultimately a reduction in plant seed set.

Pollinators face a metabolic trade‐off when foraging for pollen and nectar (Charnov, 1976; Vaudo, Patch, Mortensen, Tooker, & Grozinger, 2016) and optimal foraging theory predicts that they will maximize gain and minimize loss of energy (Charnov, 1976). Thus, pollinators may forage slowly through habitats rich in floral resources, minimizing travel distances between flower visits, and either avoid or promptly traverse florally poor habitats (Lander, Bebber, Choy, Harris, & Boshier, 2011; Pasquet et al., 2008). Moreover, pollinator forging distances have been shown to exhibit an inverse relationship with the proportion of available foraging habitat (Carvell et al., 2012). Pollinator sensitivity to the dispersion of floral resources at different spatial scales is partly influenced by traits, such as body size, that predict their mobility and capacity to forage and disperse pollen (Greenleaf, Williams, Winfree, & Kremen, 2007; Redhead et al., 2016). Given the capacity of pollinators to mediate plant gene flow, changes in foraging behavior or pollinator community composition (e.g., body size distributions) in response to variation in habitat floral resources may profoundly affect plant fitness (Vanbergen et al., 2014; Ward, Dick, Gribel, & Lowe, 2005). This may be particularly important for spatially isolated populations of uncommon plant species because increases in floral diversity might lead to greater inter‐specific plant competition for pollinators (Ghazoul, 2006) and reduce the probability of long‐distance pollen dispersal (Eckert et al., 2010).

One approach to understanding the interaction between floral community diversity and pollinator‐mediated gene flow in locally rare plant populations is to analyze plant mating patterns using highly variable molecular markers (microsatellites). This permits inference, and even direct observation, of patterns of gene movement and mating (Ashley & Dow, 1994), enabling the quantification of relatedness between plants (Ashley & Dow, 1994). The use of such molecular methods has revealed that plant populations often exhibit spatial genetic structure, where relatedness declines with distance between individuals (Loveless & Hamrick, 1984). Increased frequency of mating between close relatives within plant populations can lead to biparental inbreeding, resulting in reduced allelic diversity and greater homozygosity, which has been linked to a reduction in the fitness and long‐term survival of plants (Byers & Waller, 1999). Low allelic diversity is particularly detrimental for self‐incompatible plants whose reproduction requires allelic variation at a single locus (the ‘S‐locus’; Byers & Meagher, 1992). Although mutations can cause self‐incompatibility systems to break down, resulting in partial self‐compatibility, self‐fertilization and mating between close relatives in these plants is typically prevented (Richards, 1997). As S‐alleles are frequently lost through genetic drift, plant populations could face a reduction in compatible mates with negative implications for plant reproduction (Wagenius, Lonsdorf, & Neuhauser, 2007). Self‐incompatibility coupled with spatially structured populations may therefore render some plant species vulnerable to reductions in gene flow due to altered pollinator foraging behavior.

In this study, we investigated how the genetic connectivity and reproductive success of a locally rare and partially self‐compatible plant species was affected by habitat floral cover and the activity and richness of pollinator communities. To simulate a species occurring at low population densities, we deployed small arrays of Californian poppy (*Eschscholzia californica*) into a landscape‐scale field experiment where floral cover had been manipulated through agri‐environment planting of wildflower patches. In these experimental arrays, we measured pollinator activity, insect‐vectored pollen movement using microsatellite genotyping, seed set, and progeny viability. Based on previous observations of altered pollinator behavior in response to floral cover (Heard et al., 2007), we hypothesized that:

- Habitats supporting high floral cover will increase the activity densities and richness of pollinator species in the vicinity of experimental arrays of a partially self‐compatible plant (*E. californica*);
- The body size distribution of pollinators would be greater in florally rich habitats, reflecting the preference of *Bombus* spp. to flower species within sown wildflower patches (Carvell, Meek, Pywell, Goulson, & Nowakowski, 2007);
- Pollen movement between introduced experimental arrays of *E. californica* would be reduced in florally rich habitats, leading to pollen limitation, lower outcrossing rates, and fewer long‐distance pollination events;
- The reproductive success (seed set and progeny viability) of *Eschscholzia californica* would be reduced in florally rich habitats, reflecting a higher incidence of self‐fertilisation.

## 2 MATERIALS AND METHODS

### 2.1 Experimental site and study system

**Figure 1:**
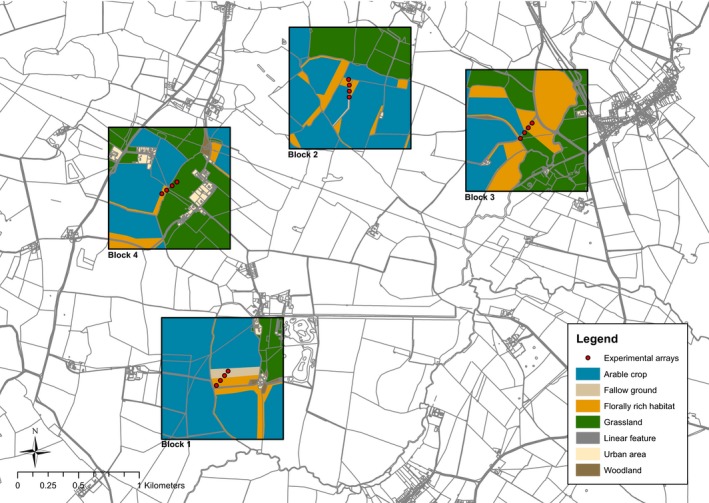
The experimental setup at the Hillesden estate, Buckinghamshire, UK. Blocks are denoted by boxes and are labeled blocks 1–4. Florally rich habitat represents all wildlife habitat options implemented under the English agri‐environment scheme

The experiment was conducted on the Hillesden estate in Buckinghamshire, UK (1°00′01′’W, 51°57′16′’N), an intensive arable farm (~1000 ha) situated on heavy clay soils with a relatively flat topography. Since 2005, a number of experimental landscape management “treatments” have been established and managed across the estate within a randomized block design. These treatments, applied to 50–60 ha replicated land parcels, comprise varying proportions (0–8% of land out of production) of a range of wildlife habitat restoration options (including pollen and nectar‐rich flower margins and wildflower patches for pollinators) under compliance with the English agri‐environment scheme (Pywell et al., 2015). Overall, these wildlife habitats comprised ~4% of the total area (Figure 1).

To test our hypotheses, we introduced the Californian poppy, *Eschscholzia californica* Cham., (Papaveraceae) (Seed source: Chiltern seeds Ltd., Wallingford, UK). Although considered naturalized in the UK (Preston, Pearman, & Dines, 2002), *E. californica* was locally absent, allowing us to unequivocally ascribe paternity in mating events. *Eschscholzia californica* is a diploid species, with a partially self‐compatible mating system, characterized by a low propensity to self‐fertilize (Wright, 1979), and thus predominantly requires insects for pollen transfer (Becker, Gleissberg, & Smyth, 2005). It possesses large, open flowers and is visited by a variety of insects from the orders: Diptera, Hymenoptera, and Coleoptera (summarized in; Cook, 1962).

In early June 2015, groups of three potted *E. californica* plants were positioned in a triangular, experimental array to simulate a locally rare plant population. Plants were separated by 1 m to prevent fertilization by direct neighbor contact. A total of sixteen arrays were introduced for a 16‐day period across four 100 ha replicate blocks (four arrays per block) separated by >500 m to minimize between block movement of insect pollinators (Figure 1). At the center of each block, four experimental arrays were placed at 50 m intervals along a 150‐m transect laid symmetrically across the boundary between an established wildflower patch (henceforth “florally rich” habitat) and bare, fallow ground (henceforth “florally poor” habitat) (Figure 1). This ensured the first two arrays on a transect were located within the florally rich habitat, and the second two arrays within the florally poor habitat. The use of agri‐environment scheme wildflower patches, sown with a common mix of 25 species, including *Trifoilum pratense*,* Centurea nigra,* and *Leucanthemum vulgare* at a rate of 37 kg/ha (Carvell et al., 2007), allowed for the standardization of florally rich treatments across the four blocks. To ensure our habitat classification was accurate, prior to the start of the experiment, we established the local floral abundance (Mean ± SE flowers/m, florally rich = 235.25 ± 42.15; florally poor = 26.25 ± 14.08) and plant diversity (Shannon Mean ± SE florally rich = 0.83 ± 0.17; florally poor = 0.28 ± 0.15) by recording all floral units within a 1m radius surrounding each experimental array (plant species list for each habitat type: Table S1).

### 2.2 Pollinator activity and species richness

Pan traps are typically deployed to describe pollinator species richness and activity densities (Westphal et al., 2008). They have also been used to provide a surrogate measure of visitation, allowing for longer periods than standard observation methods (Ricketts et al., 2008). However, this survey method has been recognized to exhibit bias (Roulston, Smith, & Brewster, 2007) because the attractiveness of pan traps depends upon habitat and landscape context (Baum & Wallen, 2011). Pollinators are less likely to encounter traps when floral resources are abundant and more likely to encounter traps when floral resources are scarce, that is, capture rates are proportional to visitation rates per unit flower area (Veddeler, Klein, & Tscharntke, 2006). We exploited this phenomenon to measure the attractiveness and pollinator activity density at our experimentally rare plant populations located within different habitats.

Pan traps comprised three water‐filled circular plastic bowls (80 × 200 mm) painted with nontoxic fluorescent paint (1 yellow, 1 blue and 1 white; UV Gear, UK) placed in the center of each array. Traps were deployed for 24 hr at each of the 16 arrays on the same day, twice weekly over the 16‐day study period (totaling four surveys). Each survey was performed in randomized order, between 0930 and 1700. Emptied traps were left *in situ* to maintain the same levels of visual attractiveness to foraging insects throughout the experiment. All insects from the main pollinator groups (Hymenoptera: Apoidea, Diptera: Syrphidae and Lepidoptera) were counted and identified to species level. In addition, given that insect pollinator body mass correlates with foraging range (Greenleaf et al., 2007) and to a lesser extent, pollen deposition (Larsen, Williams, & Kremen, 2005), we measured the intertegular span (the distance between the wing bases) of each insect from the main pollinator groups using digital calipers (given the relationship between intertegular span and body mass (Cane, 1987)). From this we determined the body size distribution of pollinator communities.

To ensure pollinators caught within pan traps could be used as a proxy for visitation, these data were calibrated by direct visitor observations on the *E. californica* plants. Pollinator visitor observations were conducted for each experimental array between 09.30 and 17.00 over four surveying days (two per week). Observations lasted for 15 min, during which every insect foraging (contacting an anther or stigma) was recorded and identified to a broad pollinator group as above.

### 2.3 Genotype analysis

*Eschscholzia californica* was grown in compost under glasshouse conditions (day: night = 20°C:15°C photoperiod light: dark = 12:12 hr). Once at seedling stage, 50 mg of fresh leaf material was removed from 95 plants and DNA was extracted from each sample following the Qiagen DNeasy 96 plant kit protocol (QIAGEN Ltd., Manchester, UK). The concentration of DNA was quantified on a spectrometer (ND8000) and subsequently diluted to 10 ng/μl. Polymerase chain reaction (PCR) was conducted using seven nonoverlapping microsatellite markers (Veliz, Gauci, & Bustamante, 2012) with fluorescent dyes attached to the forward primer (DS‐33 dye set; Applied Biosystems^™^, CA, USA). Separate PCRs were conducted for each primer set, with the exception of two primers (Ecalifdi11 and Ecalifdi1), which were successful in a multiplex PCR.

The PCR program settings were as follows: 95°C for 5 min, 35 cycles of 94°C for 30 s, 55°C (or 56°C depending upon loci) for 60 s, 72°C for 30 s, followed by a final elongation phase of 72°C for 10 min. Standard reaction conditions were as follows: 10 ng of DNA, 0.1 μl of reverse primer (20 μmol/L), and DS‐33 attached forward primer (20 μmol/L), 0.08 μl dNTPs (100 μmol/L), 0.1 μl BSA, 1 μl Buffer, and 0.1 μl Taq polymerase in a 10‐μl reaction. The PCR products were combined and visualized on a 2% agarose gel. Fragment analysis was then performed on an ABI3730 under the following conditions: 0.3 μl Liz 500 size standard, 8.7 μl HiDi formamide, and 1 μl PCR product. Alleles at all seven loci were manually scored on Genemarker V1.95, and ambiguous alleles were cloned and sequenced using TOPO^®^ TA cloning kit^®^ (Invitrogen^™^, CA, USA) to verify that they were true alleles. Following this, we selected 48 plants with distinct genotypes to be deployed at predetermined locations across the landscape (Figure 1). Where possible, plants were selected so that the three individual plants within each array were homozygous with the same allele at a selected locus. Whereas each experimental array (a triplet of plant individuals) within a block was homozygous for a different allele at this locus. This allele structure in the design allowed for verification of long‐distance pollen movement (i.e., the presence of a novel allele at the selected locus was indicative of the array from which the pollen was sourced). During initial assessments, the selected plants were shown to be polymorphic at the seven studied loci (7 loci: number of alleles, A = 2–8; observed heterozygosity, Ho = 0.083–0.75). This points toward a high diversity of S‐alleles in the base population, indicating cross‐compatibility between parent plants.

### 2.4 Pollen movement

To detect pollination events, we genotyped approximately ten progeny per plant from each of the 48 field exposed plants (Mean ± SE = 9.52 ± 0.39) using 50 mg of fresh leaf material and following protocols as above. The incidence of self‐fertilization in plants from each habitat was calculated manually by individually comparing each successfully amplified progeny against their maternal plant. If, at each of the seven loci, the progeny was a complete match for the maternal genotype, or was homozygote for one of the maternal plants alleles, it was scored as selfed. Alternatively, if any novel alleles were observed in the progeny that were not present in the maternal plant, the progeny was classified as outcrossed. Paternity was determined using Cervus 3.0.7 (Kalinowski, Taper, & Marshall, 2007), where each progeny sample was listed detailing alleles at the seven microsatellite loci, specifying the known maternal sample as well as the potential paternal samples. Here, we analyzed all progeny from within a block against all potential parents within that block. We accounted for self‐fertilization and selected for the most likely paternal parent based on a derivative of likelihood ratios; the delta score (∆), which is the difference between the likelihood score of the most likely parent and the second most likely parent (Marshall, Slate, Kruuk, & Pemberton, 1998). We only included assignments with a trio ∆ confidence (the likelihood score of a mother‐father‐offspring match) above 95%, which is classified as high confidence (Marshall et al., 1998). For all paternal assignments, we recorded which habitat, if any, the pollen had crossed together with the distance travelled.

### 2.5 Plant fitness components: seed production, germination rates, and progeny traits

All open flowers were removed from the 48 genotyped *E. californica* plants, prior to their placement in premarked locations across the landscape. They remained in the field for 16 days to ensure full anthesis of new flowers (which takes 3–4 days; Becker et al., 2005) and to allow for multiple pollination events. After this period, all fruit were tagged to ensure that only fruit development arising from the period of the field experiment were included in analyses. Plants were then collected and stored under controlled glasshouse conditions (as above) until fruit maturation. Upon maturation, tagged fruit were collected and the number of filled seeds per fruit was counted to quantify seed set per plant.

To determine whether field exposed plants were limited by pollen, we supplemented a flower from each of the 48 plants with outcrossed pollen. This involved methodically wiping four dehiscing anthers from a donor plant onto the receptive stigma of a field exposed plant with dissecting tweezers. Supplemented flowers were then covered with fine muslin to protect against accidental windborne transfer of pollen from the glasshouse air‐conditioning system. Once matured, fruit were collected and the number of seeds per fruit was counted to determine maximum seed set. The degree of pollen limitation was expressed as a ratio between the actual seed set (field exposed plants) and the potential seed set (supplemented) in each of the 48 field exposed plants.

To measure the viability of progeny from field exposed plants, 20 seeds from each of the 48 plants were sown into compost and kept under glasshouse conditions (as above). Germination was recorded daily over a 30‐day period, and any seeds which had not germinated after 90 days were recorded as nonviable. The germination success was expressed as a ratio between the number of seeds which successfully germinated against the number of seeds which failed to germinate in each of the 48 field exposed plants. Indeed, some species and populations of *E. californica* can exhibit seed dormancy (Cook, 1962), although this was found to be absent within our experimental plants (personal observation).

To further assess how reproduction by self‐fertilization affects the viability and growth traits of a partially self‐compatible plant, we performed a glasshouse experiment using 40 artificially crossed plants. On each plant, we emasculated two flowers and supplemented the first with outcrossed pollen and the second with self‐pollen. This involved methodically wiping two dehiscing anthers from a donor plant or the focal plant onto the receptive stigma with dissecting tweezers, before covering it in fine muslin. From each supplemented plant, we sowed a seed from the outcrossed fruit and from the selfed fruit (given that selfed fruits predominantly only produced one seed) into 1L pots. These were then stored under glasshouse conditions (as above). We recorded the following fitness traits: the germination rate, the duration from germination to reproductive maturity (time of first flower), together with the height (cm) and the number of buds at reproductive maturity (biomass).

### 2.6 Statistical analysis

Pollinator activity density (a proxy for visitation) and the cumulative counts of pollinator species recorded at each experimental array were modeled using generalized linear mixed models (GLMMs) with a Poisson error distribution. When analyzing the body size distribution of pollinator species caught within pan traps however, a log‐normal error distribution was instead used to account for non‐integers. Plant fitness components were similarly analyzed using GLMMs with a combination of Poisson (seed production per plant) and binomial (pollen limitation of each plant and the germination success of progeny) error distributions.

Within our models, fixed effects comprised of habitat type (florally rich/florally poor). Experimental block (Figure 1) was fitted as a random effect to account for the spatial structure of our experimental design. For pollinator activity models, additional random effects were included to account for survey date and the pollinator species, when analyzing the activity densities (64 surveys) and body size distribution (203 pollinators) of pollinators, respectively. Additional random effects for models of plant fitness components were “plant identity” for pollen limitation (42 surviving plants) and germination success (48 plants) and “fruit nested within plant” for seed production (*n *= 618) to account for variation between plants and fruit. Where present, overdispersion in the data was controlled for by fitting an observational level parameter to the random effects (Harrison, 2014). We used AIC stepwise selection to find the minimum adequate model (Burnham & Anderson, 2003) and analyzed all models using Laplace approximation. The significance of the final models was analyzed by comparison with a null model with the same random effects structure using an ANOVA. All analyses were conducted with R version ×64 (R Core Team 2013) using the lme4 package (Bates, Maechler, Bolker, & Walker, 2015).

When analyzing the effects of self‐fertilization on plant fitness traits (e.g., height), we used a combination of chi‐square contingency tables (the germination of selfed and outcrossed seeds), generalized linear models (GLMs) with a Poisson error distribution (plant height at reproductive maturity) and ANOVAs (duration to reproductive maturity and plant biomass at reproductive maturity). In both GLMs and ANOVAS, the fitness trait measured was modeled against the mating system (outcrossed or selfed) for all surviving germinated seeds (*n *= 56).

When analyzing pollen movement parameters, we used a combination of chi‐square contingency tables (the incidence of self‐fertilization modeled against the number of outcrossing events) and binomial proportion tests (the distance of pollination events, the movement of pollen across habitats of different floral covers and the movement of pollen to and from habitats of different floral covers). For the distance of pollination events, we analyzed the cumulative number of long‐distance pollination events at each distance (50, 100 and 150 m) against the total number of long‐distance (50–150 m) pollination events (*n *= 34). For the movement of pollen across habitats, we analyzed all 50 m movements where the intervening habitat varied (i.e., florally poor, a mixture of florally poor and florally rich and florally rich), against the total number of 50 m pollination events (*n *= 22). The movement of pollen to and from each habitat was similarly analyzed by comparing the cumulative counts of long‐distance pollination events (50–150 m) leaving or entering a habitat against the total number of long‐distance (50–150 m) pollination events (*n *= 34). For all models of pollen movement, we used cumulative counts across all blocks. The relationship between the number of selfing incidents and the total number of long distance movements (50–150 m) to and from each array was then analyzed against the abundance of pollinators caught in pan traps using generalized linear models with a Poisson error distribution.

## 3 RESULTS

### 3.1 Pollinator activity and species richness

**Figure 2:**
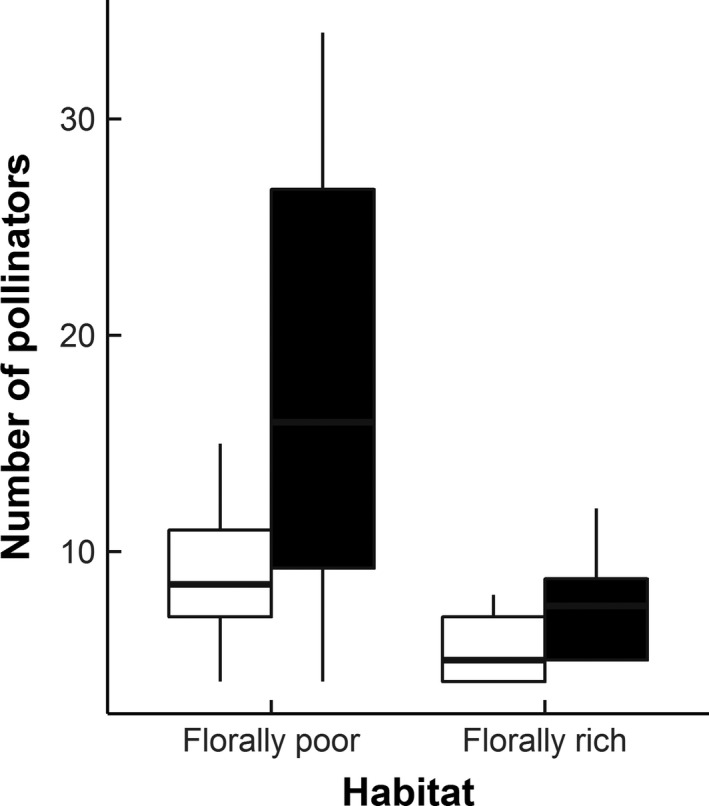
The activity densities (black boxes) and species richness (white boxes) of insects within main pollinator groups caught in pan traps within habitats differing in floral cover. Box plots represent the cumulative counts of all trapping periods, with counts averaged across each experimental array within florally poor and florally rich habitats. Bars summarize the median value (50th percentile), with boxes illustrating the upper and lower quartiles (25th and 75th percentile). Whiskers illustrate the minimum and maximum count

Considering insect taxa generally thought to be the most effective pollinators (i.e., Apoidea, Syrphidae, and to a lesser extent Lepidoptera), greater numbers were caught in pan‐traps centered on the experimental plant arrays in florally poor habitats (Mean±SE Florally rich = 7.63 ± 0.96; Florally poor = 17.75 ± 3.87; GLMM z = −3.85, *df* = 59, *p *< .0001; Figure 2). Furthermore, the species richness of these main pollinator groups was similarly higher in traps centered on plant arrays in florally poor habitats (Mean±SE Florally Poor = 9.25 ± 1.31; Florally rich = 5.5 ± 0.57; GLMM z = −2.74, *df*  = 13, *p *= .006; Figure 2) (pollinator species list from pan trap catches: Table S2). However, the body size distribution of visiting pollinators was not significantly different between florally poor and florally rich habitats (Mean ± SE Florally rich = 2.97 ± 0.13; Florally poor = 2.60 ± 0.07; *p *= .427).

The activity density of the main pollinator groups was mirrored by the overall catches of all potential pollinators (including non‐Syrphid Diptera and Coleoptera). Twice as many pollinating insects were recorded in pan traps centered on the experimental plat arrays in florally poor habitats (Mean ± SE 672.5 ± 103.14) compared to florally rich habitats (Mean ± SE 318.5 ± 56.83) (GLMM z = −4.68, *df* = 59, *p *< .0001). Non‐Syrphid Diptera and Coleoptera comprised the greatest proportion of flower visiting taxa in both habitats (Florally poor = 0.97, Florally rich = 0.98) reflecting their typically greater abundance, although their efficacy as pollinators is debated (but see Orford, Vaughan, & Memmott, 2015).

**Figure 3:**
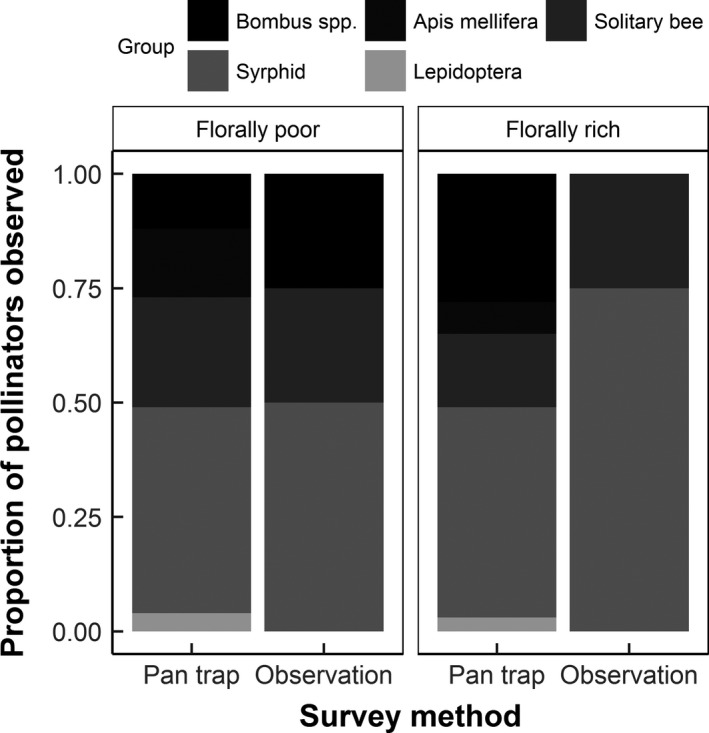
The proportion of insects within main pollinator groups observed during direct visitor observations of *Eschscholzia californica* plants and those caught in pan traps within habitats differing in floral cover

The catches of pollinators within pan traps (from the main pollinator groups: Apoidea, Syrphidae and Lepidoptera) closely reflected the proportions observed to actively visit *E. californica* (Figure 3), justifying the use of activity densities from pan traps as a proxy for actual plant visitation. Statistical analysis of these direct observations of pollinator visitation was however precluded by the sparseness of these data (total insects observed = 215 individuals).

### 3.2 Pollen movement

As expected for a partially self‐compatible species, levels of selfing were low in field exposed plants. However, the proportion of progeny that were produced by self‐fertilization was marginally greater from plants within florally rich habitats (Florally rich=15%; Florally poor = 9%; **χ**² = 3.69, *df* = 1, *p *= .055). The incidence of selfing was not, however, correlated with pollinator activity densities (*p *= .097).

**Figure 4:**
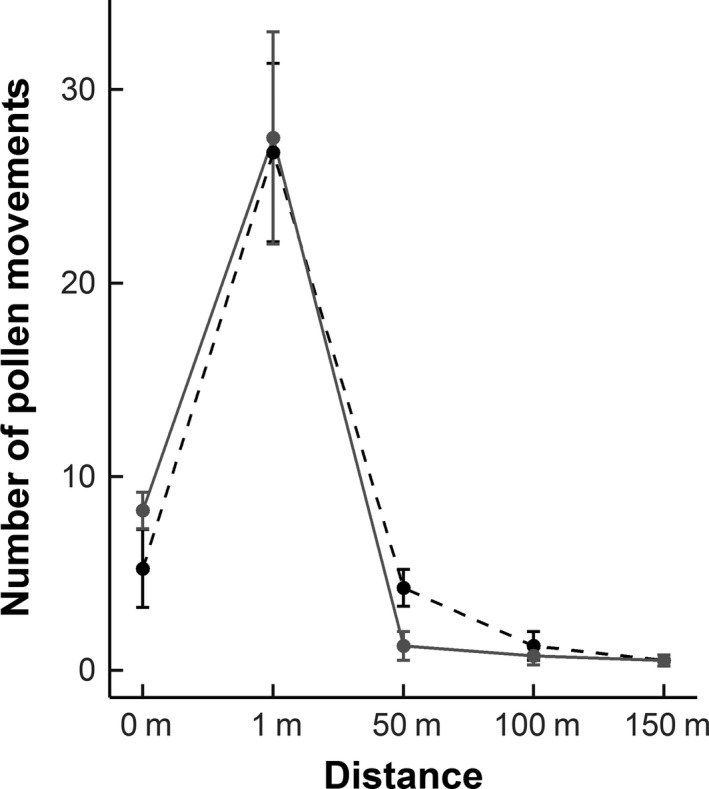
The distance of pollen movement, averaged across all blocks, from experimental arrays located within habitats differing in floral cover (self‐fertilization is denoted by 0 m). Dashed lines with open circles represent pollen movement from florally poor habitats, and solid lines with filled circles represent pollen movement from florally rich habitats

**Figure 5:**
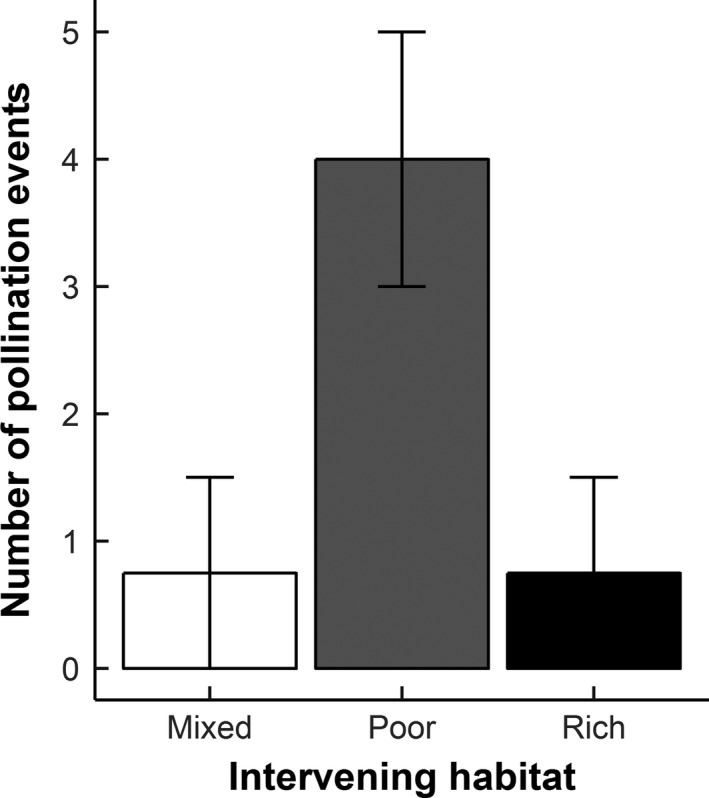
The connectivity of experimental arrays, measured by the number of long‐distance pollen dispersal events (50 m), averaged across all blocks, over habitats differing in floral cover. Mixed habitat denotes when the intervening habitat comprised of 25 m of florally rich habitat and 25 m of florally poor habitat; poor habitat denotes where the intervening habitat is comprised of 50 m of florally poor habitat and rich habitat denotes where the intervening habitat is comprised of 50 m of florally rich habitat

Paternal assignments were achieved for 300 of the 457 amplified samples, with the remainder (*n *= 157) disregarded (trio ∆ confidence score of below 95%). The greatest proportion of pollination events happened over short distances (1 m = 72%; Figure 4). We observed a number of long distance pollen movements (*n *= 34 (11% of all movements)) and of these, a significantly greater proportion travelled 50 m (65%), with fewer movements between 100 (24%) and 150 m (12%) (**χ**² = 23.65, *df* = 2, *p *< .001). These long‐distance pollen movements (50–150 m) were significantly more frequent both to (Florally rich = 32%; Florally poor = 68%; **χ**² = 7.12, *df* = 1, *p *= .008) and from (Florally rich = 29%; Florally poor =  71%; **χ**² = 9.94, *df* = 1, *p *= .002) arrays within florally poor habitats. The movement of pollen between experimental arrays was affected by the floral richness of the intervening habitat. Regarding the total number of 50 m pollination events across all blocks, pollen movement was greatest between two arrays positioned within florally poor habitats, that is, where the intervening habitat had low floral cover (Florally poor cover = 73%, a mixture of both florally poor and florally rich cover = 14% and florally rich cover = 14%; **χ**² = 23.05, *df* = 2, *p *< .001; Figure 5). Furthermore, the total number of long‐distance movements (50–150 m) to and from each array was positively correlated with pollinator activity densities (GLM z = 2.06, *df* = 15, *p *= .036).

### 3.3 Plant fitness components: seed production, germination rates, and progeny traits

**Figure 6:**
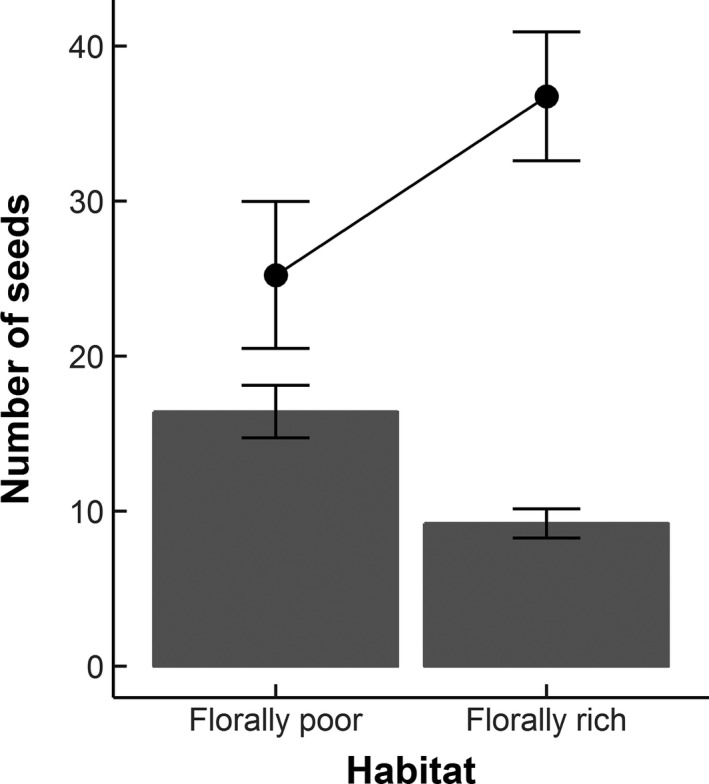
The mean number of seeds (denoted by open bars) produced by plants within habitats comprising different floral cover, together with the mean degree of pollen limitation (denoted by filled points) of these plants. Pollen limitation is illustrated here as the number of additional seeds produced by a plant after pollen supplementation (when compared to the number of seeds produced by the same plant under field conditions)

The number of fruits and seeds produced per plant was highly variable (fruit range =  4–23, seed range = 0–589). However, total seed set in arrays within florally poor habitats was 1.8–fold greater than in those within florally rich habitats (GLMM z = −1.980, *df* = 613, *p *= .048; Figure 6). Furthermore, the number of additional seeds produced by pollen supplementation was greater in florally rich habitats (GLMM z = 2.396, *df* = 38, *p *= .017; Figure 6), indicating that plants were more pollen limited in florally rich habitats.

Germination rates of progeny arising from plants located in florally rich habitats were reduced, albeit marginally (Mean±SE Florally rich = 10.67 ± 0.85; Florally poor = 12.96 ± 0.87, GLMM z = −1.940, *df* = 44, *p *= .052). Our glasshouse viability trial to quantify the implications of selfing on progeny viability showed that a lower proportion of seeds germinated when produced by self‐fertilization, compared to seeds which were a product of outcrossing (outcrossed seeds = 0.8 (*n *= 33); selfed seeds = 0.6 (*n *= 24); **χ**² = 3.91, *df* = 1, *p *= .048, phi = 0.25). However, we found no effect of self‐fertilization in *E. californica* on later stage fitness traits (time to reproductive maturity (first flower) *p *= .210; height at reproductive maturity GLMM *p *= .078; biomass at reproductive maturity *p *= .143). The negative implications of self‐fertilization were thus limited to reduced germination.

## 4 DISCUSSION

### 4.1 Habitat effects on pollinator visitation

Consistent with previous work (Veddeler et al., 2006), we found a negative association between florally rich habitats and the activity density and species richness of pollinators. Elsewhere, the abundance and richness of pollinators has been observed to increase with floral cover (Williams et al., 2015), especially where this cover is limited within the wider landscape (Heard et al., 2007). However, our results suggest that despite the increased aggregation of pollinators in habitats providing abundant, diverse floral resources, pollinator visitation, and fidelity is effectively “diluted,” which may result in lower per capita visitation and greater interspecific competition for pollination (Sjodin, 2007; Veddeler et al., 2006). Consequently, when embedded within a diverse community of co‐flowering heterospecific plants offering a variety of floral pollen and nectar, rare plant species may be unable to co‐opt pollinators (Ghazoul, 2006). In contrast, where co‐flowering, heterospecific competitors were scarce, our findings suggest that available pollinators would become concentrated, leading to potential increases in per capita visitation rates at the individual plant level (Tscharntke et al., 2012; Veddeler et al., 2006).

A diverse community of pollinators can provide niche complementarity (Pisanty, Afik, Wajnberg, & Mandelik, 2016), often leading to enhanced pollen deposition (Larsen et al., 2005) and seed production (Martins, Gonzalez, & Lechowicz, 2015). Alternatively, a high diversity of pollinators visiting diverse plant assemblages can result in an increase in heterospecific pollen deposition, which can interfere with conspecific pollination by stigma clogging (Holland & Chamberlain, 2007). The extent to which the diversity of pollinator species provides a benefit to plants is determined by the functional diversity and pollination effectiveness of communities (Perfectti, Gomez, & Bosch, 2009). Indeed, pollinator species vary in their specialization, pollen carrying behavior, and daily activity preferences, all of which affect pollination effectiveness (Martins et al., 2015; Rader, Edwards, Westcott, Cunningham, & Howlett, 2011). Furthermore, pollination effectiveness has been associated with body size, where larger pollinator species can travel greater distances (Greenleaf et al., 2007) and deposit a larger amount of pollen per visit (Larsen et al., 2005). In this study, however, we found no difference in the size distribution of pollinators between florally rich and florally poor habitats, indicating that by this measure, there was no difference in the trait structure of pollinator communities between habitats with different floral cover that could alter pollination effectiveness. Instead, pollination effectiveness may be driven by changes to the foraging behavior of pollinator communities.

### 4.2 Habitat effects on pollen movement

Consistent with previous studies, our findings indicate that pollen movement between local populations was strongly affected by the floral composition of a habitat (Dyer, Chan, Gardiakos, & Meadows, 2012; Lander et al., 2011). Pollen movement between experimental arrays (50 m) was greater when the surrounding and intervening habitat comprised livestock grazed grassland or fallow ground with low richness of floral resources. In addition, we found very few pollination events between arrays separated by habitats of high floral cover or those with heterogeneous intervening habitats (i.e., a mixture of habitats comprising high and low floral cover). These results are consistent with our hypothesis that the foraging behavior of pollinator communities is highly determined by habitat composition. This higher level of pollen movement between populations in florally poor habitats supports research which shows pollinators to conform to the weighted line foraging principle when encountering heterogeneous landscapes (Lander et al., 2013). This principle assumes that pollinators will occupy optimal foraging habitat until resources are depleted, thus making short, energy efficient, movements between flowers. Conversely, pollinators are expected under this principle to move greater distances within habitats that are nutritionally suboptimal (Lander et al., 2013). By altering the insect‐mediated connectivity between plant populations, the weighted line foraging strategy will have implications for genetic exchange and the genetic diversity of rare plant populations.

The floral cover of the surrounding habitat greatly affected the distance of pollen movement with plants in florally poor habitats subject to more long‐distance pollination events than those in florally rich habitats. We further show this to be positively correlated with activity density of pollinators. From this, we can infer that pollinators were following optimal foraging expectations, where movement reflects energy efficient behavior. Indeed, we show that in both habitats, the majority of pollen movement was localized (1 m). Of the long‐distance pollination events, a greater proportion were between plants separated by 50 m, with fewer between distances of 50–150 m. This pattern is consistent with a wealth of research indicating that although capable of travelling large distances (Hagler, Mueller, Teuber, Machtley, & Van Deynze, 2011), pollinators predominantly travel considerably shorter distances (Rader et al., 2011), remaining in localized resource patches (Pasquet et al., 2008). This results in a distance decay distribution of pollen movement (Matter, Kettle, Ghazoul, Hahn, & Pluess, 2013), suggesting that between block movement (>500 m) in this experiment would be minimal. In spatially genetically structured plant populations, reduced long‐distance pollination events, particularly in florally rich habitats, will result in a higher frequency of mating between close relatives. As a consequence, self‐incompatible and partially self‐compatible plants will suffer from increased biparental inbreeding and a reduction in compatible mates (Turner, Stephens, & Anderson, 1982). This will negatively impact plant seed set and viability (Ward et al., 2005), together with the adaptive potential and consequently the long‐term survival of rare plant populations (Etterson, 2004).

### 4.3 Implications for plant reproductive success

Reductions in the activity densities and richness of pollinator species in florally rich habitats reflect the increased pollen limitation and reduced individual plant reproduction observed within experimental arrays located in florally rich habitats. Pollen limitation has been related to competition for pollinator visitation, with similar results observed in response to an increase in diversity (Vamosi, Steets, & Ashman, 2013) or density (Jakobsson, Lazaro, & Totland, 2009) of co‐flowering plants. Low pollen receipt, a cause of pollen limitation, can result either in an increase in self‐fertilization (Kalisz, Vogler, & Hanley, 2004), or in the case of self‐incompatible or partially self‐compatible plants, where it is particularly detrimental, a direct reduction in seed production (Wagenius et al., 2007). Given the limited duration of stigma receptiveness, the ability of a plant to attract pollinators is therefore important for both pollen receipt and seed production (Bernhardt, Mitchell, & Michaels, 2008).

As well as the supply of pollen, the quality of pollen is also critical to plant reproduction and fitness. Pollen quality refers to both the deposition of heterospecific pollen, which can result in physical or chemical inhibition of seed set (Holland & Chamberlain, 2007; Kanchan & Jayachandra, 1980) and to the genetic relatedness of pollen, which can lead to inbreeding depression (Fischer, Hock, & Paschke, 2003). Our findings indicate that, through alterations to pollinator visitation and subsequent reductions in pollen receipt, florally rich habitats can promote higher levels of self‐fertilization. Further, given reduced germination rates in progeny from plants in florally rich habitats and the negative relationship observed between germination and self‐fertilization, results are indicative of higher rates of self‐fertilization then detected by microsatellite analysis. Reproduction by selfing in self‐incompatible or partially self‐compatible plants can have a negative impact on the fitness of progeny, shown in this study through a reduction in germination rates. These findings are consistent with previous research where self‐fertilization in self‐incompatible plants resulted in inbreeding depression with negative implications for plant fitness (Bellanger, Guillemin, Touzeau, & Darmency, 2015). However, in contrast to previous studies (Thiele, Hansen, Siegismund, & Hauser, 2010), reductions in germination did not translate into negative impacts on late fitness traits (e.g., time to reproductive maturity) of surviving plants. This suggests that the immediate effects on population persistence would be due more to changes in vital rates than trait differentiation.

### 4.4 Implications for the conservation of rare plants

Rarity in plants can be driven by biological or anthropogenic factors and is often characterized by populations comprising low genetic variation together with restrictions in size, local abundance, geographical range, and/or habitat specificity (Espeland & Emam, 2011). In this study, by simulating rare plant populations, we show that restrictions in a plant's population size, over the longer term, could lead to an Allee effect, whereby increases in mating between close relatives, coupled with higher self‐fertilization rates further reduces genetic variation and ultimately, increases the risk of local extinction (Etterson, 2004). We suggest that conservation efforts for plants facing conditions associated with rarity may benefit from focus on enhancing visitation and movement of pollinators between conspecifics. This could be achieved through a combination of: i) increasing the competitive advantage of plant populations (e.g., increasing a plant's population size; Mayer et al. 2012), ii) managing surrounding habitats to enhance facilitation of pollinators to plant populations (e.g., introducing co‐flowering species which have complementary phenotypes; Ghazoul, 2006), and iii) reducing the distance between conspecific populations (Van Rossum & Triest, 2010).

## 5 CONCLUSION

Our findings show that habitat context mediates plant–pollinator interactions and alters the reproduction of rare plant populations. In florally rich habitats, rare plant populations are at a competitive disadvantage for pollinator visitation when faced with more abundant co‐flowering heterospecific plants. Consequently, rare plant populations in these habitats suffer from increased rates of self‐fertilization, limited pollen movement, and reduced reproductive success. The implication is that plant populations dependent on insect pollinators may become less connected and more genetically depauperate when located in florally rich habitats, increasing the risk of genetic drift and extinction. Such an effect may hold for not only rare plants but also plants that are widespread but occur at low frequency within the environment.

Indeed, pollinator behavior has been observed to alter in relation to landscape context at spatial scales related to foraging capacity (Steffan‐Dewenter, Munzenberg, Burger, Thies, & Tscharntke, 2002). Although not touched upon here given the small scale of the study, this might be expected to affect plant and pollinator interactions at the habitat level and therefore warrants future study.

## STATEMENT OF AUTHORSHIP

All authors contributed substantially to the design and planning of the experiment, TME conducted the experiment, collected the data, and performed the analysis. TME, SC, and RE performed the paternity analysis, TME wrote the first draft of the manuscript. All authors contributed substantially to revisions and gave final approval for publication.

## DATA ACCESSIBILITY

Data supporting results is archived in the NERC Environmental Information Data Centre (EIDC), and the data DOI will be included at the end of the article.

## CONFLICT OF INTEREST

None declared.
